# Integrated in situ gas stripping–salting-out process for high-titer acetone–butanol–ethanol production from sweet sorghum bagasse

**DOI:** 10.1186/s13068-018-1137-5

**Published:** 2018-05-10

**Authors:** Hao Wen, Huidong Chen, Di Cai, Peiwen Gong, Tao Zhang, Zhichao Wu, Heting Gao, Zhuangzhuang Li, Peiyong Qin, Tianwei Tan

**Affiliations:** 1 National Energy R&D Center for Biorefinery, Beijing University of Chemical Technology, No. 15 Beisanhuan East Road, Chaoyang District, Beijing, 100029 People’s Republic of China; 2 College of Life Science and Technology, Beijing University of Chemical Technology, Beijing, 100029 People’s Republic of China; 3 College of Chemical Engineering, Beijing University of Chemical Technology, Beijing, 100029 People’s Republic of China; 4 Center for Process Simulation & Optimization, Beijing University of Chemical Technology, Beijing, 100029 People’s Republic of China

**Keywords:** ABE fermentation, Gas stripping, Salting-out, In situ product recovery

## Abstract

**Background:**

The production of biobutanol from renewable biomass resources is attractive. The energy-intensive separation process and low-titer solvents production are the key constraints on the economy-feasible acetone–butanol–ethanol (ABE) production by fermentation. To decrease energy consumption and increase the solvents concentration, a novel two-stage gas stripping–salting-out system was established for effective ABE separation from the fermentation broth using sweet sorghum bagasse as feedstock.

**Results:**

The ABE condensate (143.6 g/L) after gas stripping, the first-stage separation, was recovered and introduced to salting-out process as the second-stage. K_4_P_2_O_7_ and K_2_HPO_4_ were used, respectively. The effect of saturated salt solution temperature on final ABE concentration was also investigated. The results showed high ABE recovery (99.32%) and ABE concentration (747.58 g/L) when adding saturated K_4_P_2_O_7_ solution at 323.15 K and 3.0 of salting-out factor. On this condition, the energy requirement of the downstream distillation process was 3.72 MJ/kg of ABE.

**Conclusions:**

High-titer cellulosic ABE production was separated from the fermentation broth by the novel two-stage gas stripping–salting-out process. The process was effective, which reduced the downstream process energy requirement significantly.

**Electronic supplementary material:**

The online version of this article (10.1186/s13068-018-1137-5) contains supplementary material, which is available to authorized users.

## Background

Biobutanol was a kind of alternative fuel and an important building block in chemical industry [1]. The production of biobutanol by acetone–butanol–ethanol (ABE) fermentation was still difficult and challengeable, though it was attractive in replacing fossil fuels and solving the environmental problems [2]. In recent years, the production of biobutanol from renewable lignocellulosic biomass materials attached much attention, as it did not compete with food supply by utilization and conversion of the cheap polysaccharides including cellulose and hemicelluloses [3].

However, low productivity and low concentration of ABE were caused by the severe inhibition of ABE production and toxic components in the biomass hydrolysate [4]. One of the effective ways to solve the above obstacles is integrating the ABE fermentation process with in situ product recovery (ISPR) [1, 4]. Among different types of the alternative solvents recovery techniques, gas stripping was favored by researchers, as it is easy to operate and no harm to the culture [5]. However, limited by the vapor–liquid equilibrium, the gas stripping efficiency for ABE separation was relatively low [6]. More importantly, solvents production in the fermentation broth was difficult to be separated completely in the gas stripping process [7]. To recover butanol with higher purity and decrease the energy requirement in the solvents separation process, two-stage separation processes including two-stage gas stripping [8], gas stripping–pervaporation [9, 10], and solvent extraction–gas stripping [11] were developed and coupled with the ABE fermentation processes. However, drawback was that the second-stage separation unit of the above two-stage separation processes was ineffective. Except for the energy requirement, there was still a large amount of water in the outputting ABE solution. In addition, distillation was required for further ABE solvents dehydration [12].

Salting-out was another effective method for ABE separation. An upper organic phase with low water content and high-titer solvents was obtained [13]. However, salting-out was limited by the large amount of salt requirement and the difficulty in salt recovery [14]. In addition to this, the salting-out process was difficult to be integrated with fermentation process for ISPR because of the high osmotic pressure to strains. Therefore, salting-out was unable to improve the ABE fermentation performance by ISPR, though it was always effective [15].

In this study, aiming to improve the ABE fermentation performance and to improve the energy-intensive downstream processes, two-stage gas stripping–salting-out process was integrated with fed-batch ABE fermentation using sweet sorghum bagasse (SSB) as raw material. In this process, K_4_P_2_O_7_ and K_2_HPO_4_ were used to separate ABE from the gas stripping condensate. Since the volume of the gas stripping condensate was far smaller than that of the fermentation broth, the salt requirement was much lower than that of the conventional salting-out process treating the ABE fermentation broth directly (e.g., the ABE concentration in the condensate of gas stripping in the current work was 143.6 g/L. By contrast, the ABE concentration in the lignocellulosic hydrolysate without gas stripping was less than 12.2 g/L [10]. Thus, for given amount of ABE products, the volume of the gas stripping condensate was above 10 times lower than that of the batch fermentation broth. Correspondingly, when salting-out the solvent products from the aqueous fraction, the salt requirement based on the fermentation broth was also > 10 times of the process using gas stripping condensate). Hence, the novel integration process is effective for high-titer ABE production.

## Methods

### Chemicals and raw materials

All the chemicals used through the experiments were of analytical grade and were purchased from Beijing Chemical Works. The cellulase was purchased from KDN biotech group, China. The cellulase activity was 60 ± 5 FPU/mL.

The sweet sorghum stalks were kindly provided by Prof. Guiying Li from the Chinese Academy of Agricultural Science, and the materials were harvested in October 2015 on the experimental field in Shunyi district, Beijing, China. After squeezing process and being dried out at 105 °C overnight, the SSB was stored at − 20 °C in an oxygen-free (filled by N_2_) plastic bag.

The SSB was divided into two parts. One part was cut into chips (2–3 cm in length) and was used as the carrier for cells’ immobilization according to the method in our previous work [16]. The other part was milled into 40–60 meshes and was used as the raw material for saccharification and bioconversion.

### Strains and fermentation medium

*Clostridium acetobutylicum* ABE 1201 was stored in our lab and was used in the current research. The seed medium was similar to the previous report [17] which consisted of 70 g/L of glucose, *p*-aminobenzoic acid (1 mg/L), biotin (0.01 mg/L), and minerals (0.01 g/L of MnSO_4_ and FeSO_4_, 1 g/L of KH_2_PO_4_ and K_2_HPO_4_, 0.2 g/L of MgSO_4_, and 2.2 g/L of ammonium acetate). Nitrogen was purged by the autoclaved (121 °C for 20 min) medium to construct the anaerobic environment. And the fermentation was carried out at 37 °C without pH control and stirring.

### SSB pretreatment and enzymatic hydrolysis

Alkaline pretreatment was applied according to our previous work [10]. 2% (w/v) of NaOH was mixed with the SSB under the solid-to-liquid ratio of 1:10. Then, the slurry was maintained at 120 °C for an hour. After that, the solid fraction was separated by vacuum filtration and was washed by deionized water until the pH of the recovered bagasse decreased to ~ 7. The dry pretreated SSB was mixed with 0.01 M H_3_PO_4_/KH_2_PO_4_ buffer (pH 4.8) under the solid-to-liquid ratio of 10% (w/v). Then, 20 FPU/g (of pretreated bagasse) cellulase was mixed with the buffer. The stirring rate was 180 rpm and the temperature was kept at 50 °C. After 20 h of batch hydrolysis, 5% (w/v) of the pretreated SSB was added into the bioreactor accompany with 15 FPU/g of cellulase. Similarly, additional pretreated SSB solid and cellulase were routinely added into the bioreactor once the sugars’ productivity was slowed down. After 100 h of hydrolysis, the enzymatic hydrolysate was separated, and the pH of the broth was adjusted to ~ 7 by ammonium hydroxide. Then, the hydrolysate was vacuum concentrated until the sugars’ concentration was above 500 g/L. The concentrated SSB hydrolysate was directly used as the fermentation medium without detoxification and nutrients’ supplementation.

### Experimental setup

**Fig. 1:**
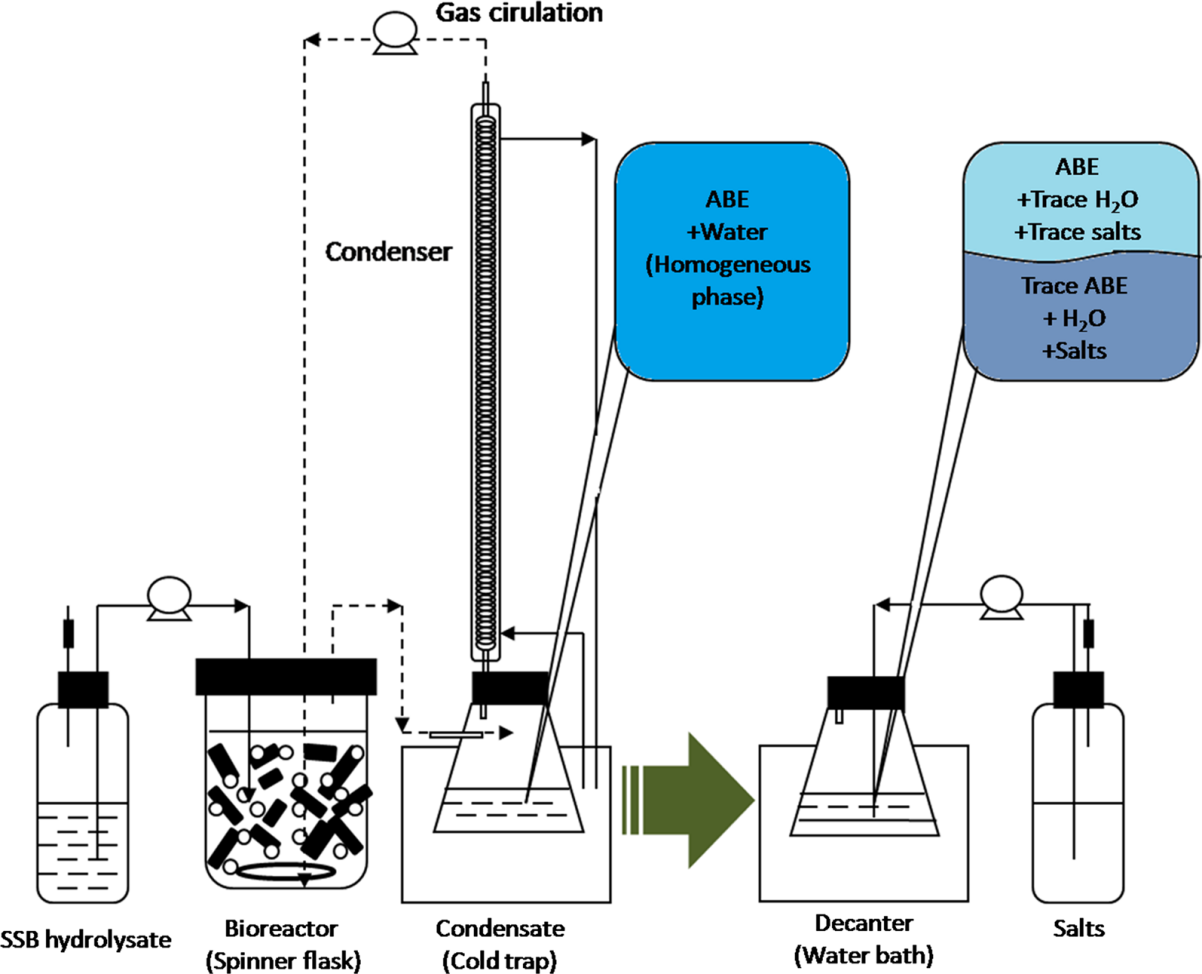
Experimental setup of the integrated ABE fermentation system with gas stripping–salting-out unit using SSB as carrier and substrate. K_4_P_2_O_7_ and K_2_HPO_4_ were used for the phase separation of the gas stripping condensate

As shown in Fig. 1, the simplified ABE fermentation–gas stripping system was similar to our previous study [17], which was connected with the salting-out unit. A 2 L bioreactor with a working volume of 1.5 L was not only used as the cells immobilized bioreactor, but also treated as the spinner flask for gas stripping. The SSB was used as the immobilized carrier, while the enzymatic hydrolysate of SSB was used as the feedstock for ABE production. Batch fermentation was carried out using the diluted SSB hydrolysate with ~ 60 g/L of initial fermentable sugars (mainly consisted of glucose and xylose). The inoculation size was 10% (v/v) and the SSB dosage rate was 2% (w/v). After 48 h of inoculation, the gas phase of the bioreactor was cycled by a peristaltic pump (under a speed of 2 L/min). In addition, the cycled gas was passed through the spiral condenser (30 × 400 mm) maintained at − 5 °C. The peristaltic pump for recycling the carrier gas was tuned on/off at each 12 h. Condensate was collected in a conical flask of 250 mL. Concentrated SSB hydrolysate was pumped into the bioreactor when the residual sugars’ concentration was below ~ 10 g/L, returning the sugar concentration back to 30–50 g/L. The ABE fermentation–gas stripping unit was ended after 180 h of fermentation period. The condensate from each cycle was collected and mixed at the end of the fermentation.

At the end of fermentation, the final gas stripping condensates collected were mixed well and were equally separated into tubes for evaluating the salting-out performance. Each group was carried out in triplicate. K_4_P_2_O_7_ or K_2_HPO_4_ was dissolved in deionized water until they were saturated at the given temperature (298.15, 310.65, and 323.15 K). The salting-out factor was defined as the ratio of the saturated salt volume and that of the ABE solution. Saturated salt solutions were added into the gas stripping condensate at certain salting-out factor (the solubility of K_4_P_2_O_7_ or K_2_HPO_4_ at given temperature was tested in laboratory. Results are shown in Additional file 1: Figure S1). After that, the system was shaken thoroughly and then settled for 12 h at 298.15 K. After the liquid–liquid equilibrium achieved, samples were collected from the organic phase and the aqueous phase for later analysis. The recovery (*R*) can be calculated from:

1 $$R = \frac{{V_{1} C_{1i} }}{{V_{0} C_{0i} }}$$

where *V*_1_ was the volume of the organic phase, *V*_0_ was the volume of the gas stripping condensate added, *C*_1*i*_ represented the concentration of *i* in the organic phase, and *C*_0*i*_ represented the concentration of *i* in the gas stripping condensate. *i *= 1, 2, 3, 4 represented ethanol, acetone, butanol, and total ABE, respectively.

### Analysis

ABE in bioreactor, gas stripping condensate, and the organic/aqueous phase of the salting-out systems as well as the organic acids was measured by gas chromatograph (GC-2010, Shimadzu, Japan) that equipped with an FID and a packed column (Porapack Q, 80/100 mesh). The concentrations of glucose and xylose in the enzymatic hydrolysate and the bioreactor were determined by a high-performance liquid chromatography that equipped with an RID and an Aminex HPX-87H column (Bio-Rad, USA). 0.005 M H_2_SO_4_ was used as the mobile phase. The method provided by US National Renewable Energy Laboratory (NREL) was used for analysis of the SSB composition [18].

The analysis of salt recycling energy cost and the energy requirement of the downstream distillation process that feeding the organic phase after gas stripping–salting-out system were simulated by UNISIM based on the NRTL model. Sequential modular approach was applied with a convergence tolerance of 1E-8.

## Results

### Fed-batch fermentation integrated with intermittent gas stripping

Effect of ABE fermentation coupled with gas stripping using sweet sorghum juice as substrate was well studied [19, 20]. However, to our best knowledge, no researches have been utilized SSB hydrolysate as substrate for the second-generation ABE production with process intensified by gas stripping. Sweet sorghum bagasse, the raw material for ABE fermentation, contained 37.1 ± 2.4% of glucan, 18.2 ± 1.6% of xylan, and 24.9 ± 2.5% of klason lignin. After alkaline pretreatment, ~ 57% of the solid fraction was recovered by filtration. The main compositions of the pretreated SSB were: 57.9 ± 3.5% of glucan, 19.8 ± 2.7% of xylan, and 3.7 ± 0.8% of klason lignin. The vacuum evaporation after fed-batch enzymatic hydrolysis and liquid–solid separation was conducted to achieve high-titer fermentable sugars’ concentration in substrate.

**Fig. 2:**
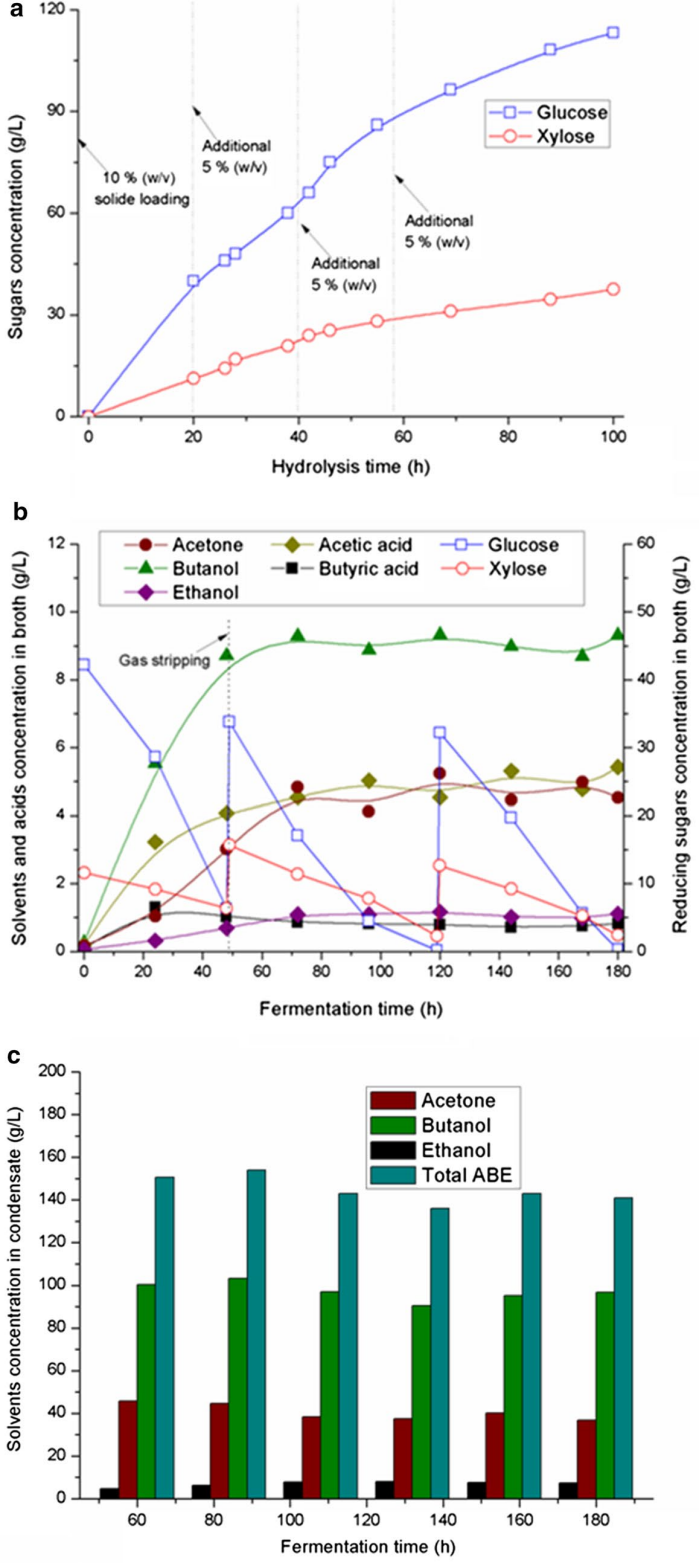
Fed-batch fermentation coupled with intermittent gas stripping for in situ ABE recovery. SSB was used as the raw material and the carrier for cells immobilization. The gas stripping unit was turned on and off for each 12 h of period. **a** Fed-batch enzymatic hydrolysis of the alkaline pretreated SSB. The concentrated enzymatic hydrolysate was used as the substrate for ABE production; **b** kinetics of solvents, acids and reducing sugar concentration remained in the bioreactor; **c** time course of ABE concentration in condensate of gas stripping unit. 36–46 mL of condensates was generated after each gas stripping period (36.2, 46.1, 41.2, 39.7, 41.1, and 44.5 mL were obtained after 60, 84, 108, 132, 156, and 180 h of inoculation, respectively)

As can be seen from Fig. 2a, after 100 h of hydrolysis, 113.2 g/L of glucose and 37.5 g/L of xylose were generated from total ~ 25% of the pretreated SSB. The hydrolysate, which contained 392.3 g/L of glucose and 126.4 g/L of xylose, was used as the substrate for subsequent fed-batch fermentation. Figure 2b shows the kinetics of ABE, acidic by-products, and reducing sugar concentrations in the fermentation broth in the overall 180 h of fermentation. Additional substrate was loaded into the bioreactor at 48 and 120 h, respectively. 153.7 g/L of the mixed sugar in the SSB hydrolysate was utilized by Clostridia. The average productivities of ABE and butanol were 0.23 and 0.15 g/L h, respectively. In addition, the yields of ABE and butanol were 0.32 and 0.2 g/g, respectively. These results were a little lower than the process using glucose as substrate (0.34 g/g for ABE and 0.2 g/g for butanol) [12]. It might be attributed to the inhibition of phenol and organic acids in the SSB hydrolysate [21]. Besides, complex pentose phosphate pathway of Clostridia for xylose utilization also leads to the decrease of solvents yield [2].

As shown in Fig. 2c, 36.8–45.7 g/L of acetone, 90.4–103.2 g/L of butanol, and 4.6–8.1 g/L of ethanol were tested in the gas stirring condensates. After mixing the condensates from each batches, the final ABE mixture containing 41.1 g/L of acetone, 95.1 g/L of butanol, and 7.4 g/L of ethanol (143.6 g/L of ABE) was obtained (the volume was 248.8 mL). It should be noted here that there was no obvious phase separation in the mixture, because there were low ABE concentration in the gas stripping condensate, which was different with the phenomenon in the previous literatures [19, 22]. It was because acetone, acted as an amphiphilic component, helped to enhance the miscibility of butanol and water in the gas stripping condensate [23]. A permeates under homogeneous phase with high ABE concentration was also obtained after pervaporation in our previous study [24]. In the following experiment, this ABE was further concentrated by a second-stage separation based on salting-out technique.

### Salting-out the high-titer ABE production from gas stripping condensate

#### Effect of salting-out agent temperature

The solubility properties of K_4_P_2_O_7_ and K_2_HPO_4_ in water were sensitive to the temperature of salt solution [25, 26]. K_4_P_2_O_7_ and K_2_HPO_4_ behaved higher solubility accompany with the increase of temperature. Besides, the different salt concentrations also have a significant effect on the liquid–liquid equilibrium [27], and further affected the separation behaviors [26–29]. The saturated K_4_P_2_O_7_ and K_2_HPO_4_ solutions at different temperatures were added into the gas stripping condensate for the second-stage ABE separation, respectively.

**Fig. 3:**
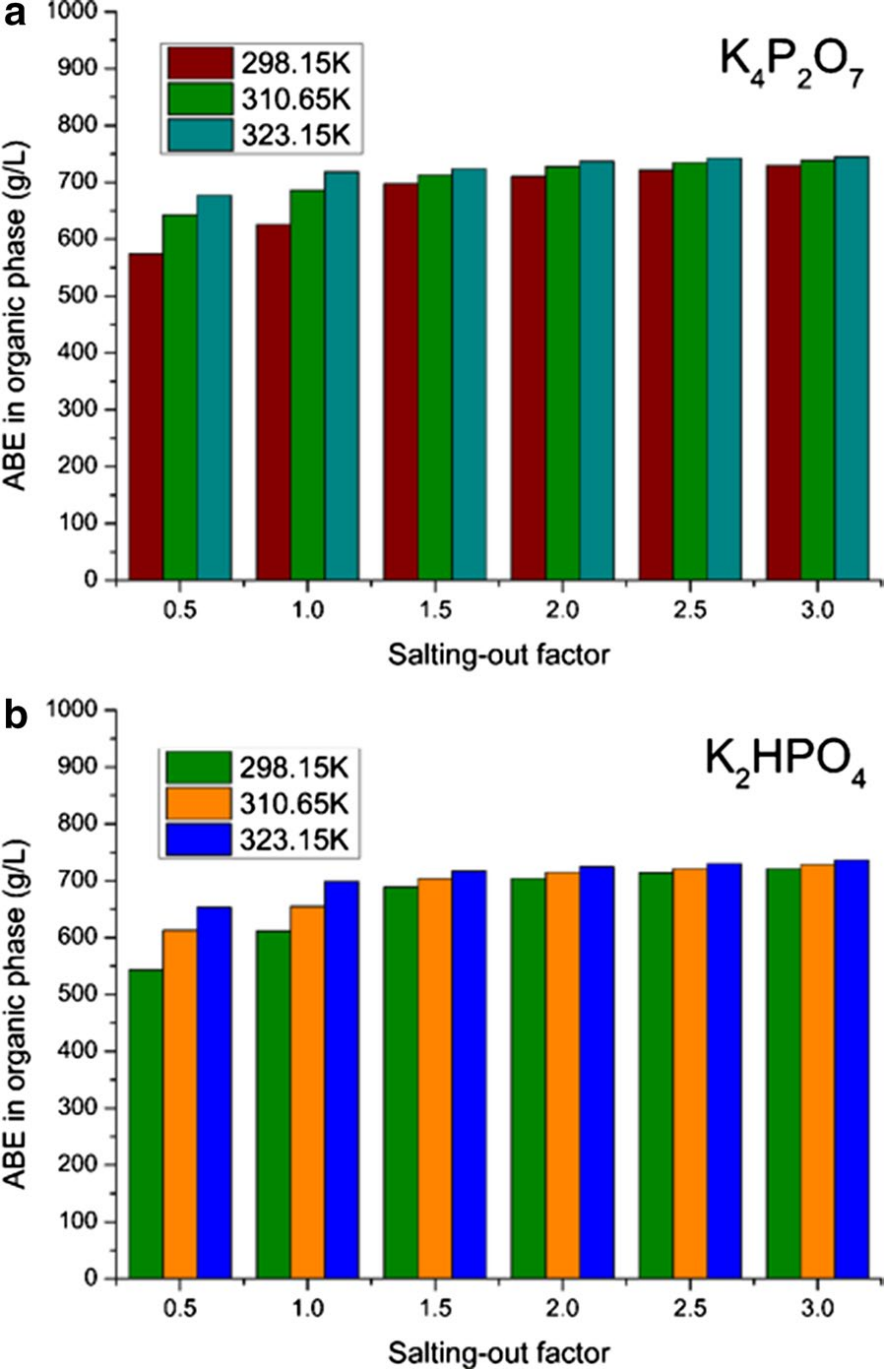
Effect of salting-out agent temperature on ABE accumulation in the organic phase. **a** K_4_P_2_O_7_; **b** K_2_HPO_4_

After the addition of salting-out agents, clear phase interfaces occurred between the organic phase and the aqueous phase in all the systems. In accordance with the previous results [13], the higher salting-out agent temperature encouraged to achieve higher ABE concentration in the organic phase. As shown in Fig. 3a, at a given saturated K_4_P_2_O_7_ solution temperature, ABE titer in organic phase increased with the increase of the salting-out factor. Similarly, the salt solution temperature also showed positive correlation to the ABE concentration in the organic phase at constant salting-out factor. More specifically, adding the saturated salt solution at 298.15 and 323.15 K, 625.87 and 718.21 g/L of ABE was detected, respectively (under the salting-out factor of 1.0).

For the K_2_HPO_4_ case, similar trend was also obtained (Fig. 3b). There were 611.42 and 698.22 g/L of ABE in the organic phase, respectively. However, in comparison with more significant effect of K_4_P_2_O_7_, the ABE obtained in the organic phase after adding K_2_HPO_4_ was less sensitive to temperature. It was due to the distinction of the anions in K_2_HPO_4_ and K_4_P_2_O_7_ solutions. As the salting-out agents provided abundant charged ions to attract water molecules [30], the agents with higher salt content were more effective to the solvents recovered. Hence, with higher solubility, the saturated K_4_P_2_O_7_ solution showed better salting-out performance compared with the groups based on K_2_HPO_4_. All in all, the saturated K_4_P_2_O_7_ solution at 323.15 K was selected for high-titer ABE concentration in the second-stage process.

#### ABE in the aqueous phase after salting-out

**Fig. 4:**
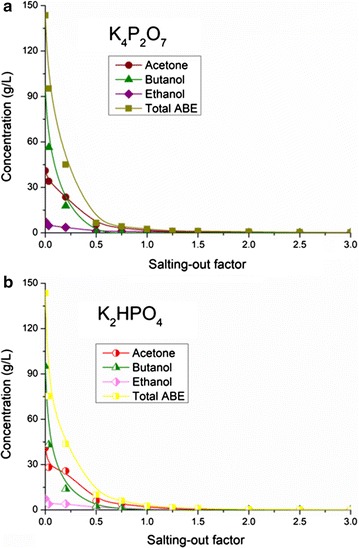
Kinetics of ABE concentrations in the aqueous phase to the salting-out factors. **a** K_4_P_2_O_7_; **b** K_2_HPO_4_

The behavior of the ABE concentrations in the aqueous phase against the salting-out factor is shown in Fig. 4. For the K_4_P_2_O_7_ groups, a clear phase interface was obtained when the salting-out factor was above 1/30. ABE concentrations decreased significantly accompany with the increase of the salting-out factor. Compared with the slight reduction of ethanol and acetone concentrations, butanol concentration dropped sharply with the increasing salting-out factor. It was attributed to the lowest polarity index of butanol in the three components [butanol (4.0) < acetone (5.1) < ethanol (5.2)], which was more prone to phase separation. Specifically, 34.02 g/L of acetone, 56.39 g/L of butanol, and 4.82 g/L of ethanol (total ABE of 95.23 g/L) were tested in the aqueous phase at the salting-out factor of 1/30. By contrast, only 0.15 and 0.08 g/L of ethanol and acetone were obtained at the salting-out factor of 3.0 (total ABE of 0.23 g/L), respectively. In addition, the residual butanol in the aqueous phase was below 0.01 g/L when the salting-out factor was above 0.5. These phenomena were generally consistent to the results in the previous works [22]. Therefore, butanol could be separated from the gas stripping condensate effectively by salting-out.

The salting-out effect on ABE concentrations using saturated K_2_HPO_4_ solution is shown in Fig. 4b. The results were similar to those in Fig. 4a. Total ABE of 75.3 g/L was determined at the salting-out factor of 1/30. Compared with the groups of K_4_P_2_O_7_, the results in the K_2_HPO_4_ groups were better for organics separation. However, the phenomenon was not inconsistent to the sequence of salting-out ability [29]. It was due to the different volumes of organic phase and aqueous phase. Usually, the volume of the aqueous phase in the K_2_HPO_4_ groups was a little larger than that of the K_4_P_2_O_7_ groups, because K_4_P_2_O_7_ had better dehydration ability than K_2_HPO_4_ [13]. That means the aqueous phase in K_2_HPO_4_ groups had lower ABE concentrations. When the salting-out factor raised to 3.0, 0.16 g/L of ethanol and 0.19 g/L of acetone (total 0.35 g/L of ABE) were remained in the fermentation broth.

The ABE contents in the aqueous phase using K_4_P_2_O_7_ and K_2_HPO_4_ are compared in Additional file 1: Figure S2. The salting-out effect on ethanol separation was not obvious compared with other fractions (Additional file 1: Figure S2a). Because of the relatively low ethanol concentration in the gas stripping condensate and the weaker salting-out ability of K_2_HPO_4_, it appeared that obvious lag phase arose in the initial stage after adding saturated K_2_HPO_4_ solution. Besides, with the salt concentration rising continuously, the slope of the residual ethanol became gentler. As for acetone, a slight lag phase was still obtained in the system based on K_2_HPO_4_ (Additional file 1: Figure S2b). In contrast to the gentle trends of ethanol and acetone, the decreasing curve of butanol was steep (Additional file 1: Figure S2c). It might be caused by the lower polarity indexes of butanol. When the salting-out factor reached 0.75, 0.91 g/L of butanol was detected in the K_2_HPO_4_ groups. As compared, butanol was not detected in the K_4_P_2_O_7_ groups. From the perspective of total ABE, the increasing dosage of salt solutions would contribute to the ABE separation and the solvents were almost recovered in the organic phase completely (Additional file 1: Figure S2d).

#### ABE recovery in the organic phase after salting-out

**Fig. 5:**
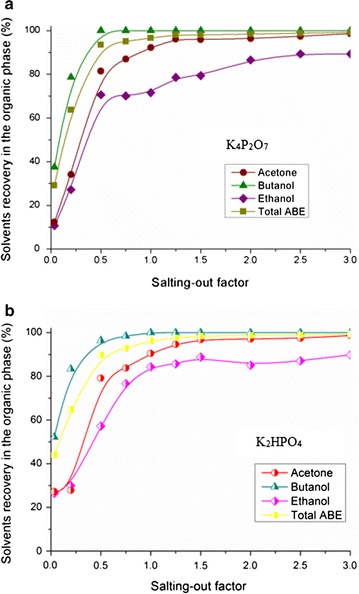
Kinetics of ABE recoveries in the organic phase to the salting-out factors. **a** K_4_P_2_O_7_; **b** K_2_HPO_4_

The effect of salt types on ABE recovery in the organic phase was further evaluated (Fig. 5). At the certain salting-out factor, the sequence of ABE recoveries was *R*_ethanol_ < *R*_acetone_ < *R*_butanol_, and this trend was generally consistent to the polarity indexes. In Fig. 5a, 86.95% of acetone, almost 100.00% of butanol and 70.09% of ethanol (95.03% of total ABE) were recovered, respectively, at the salting-out factor of 0.75 using saturated K_4_P_2_O_7_ solution. In comparison with the ABE recovery based on K_4_P_2_O_7_, the ABE recoveries of the K_2_HPO_4_ group were just 83.79% for acetone, 98.39% for butanol, 65.02% for ethanol, and 92.83% for total ABE (Fig. 5b). These results were in agreement well with the previous investigation that proved the sequence of salting-out ability: K_4_P_2_O_7_ > K_2_HPO_4_ [29], which might be attributed to the Gibbs free energy of hydration of the ions (∆_hyd_*G*) [31]. In addition, at the salting-out factor of 3.0, 99.32 and 99.29% of total ABE could be recovered in the K_4_P_2_O_7_ and K_2_HPO_4_ groups, respectively.

In Additional file 1: Figure S3, the recoveries of ethanol, acetone, butanol, and total ABE were also compared using K_4_P_2_O_7_ and K_2_HPO_4_ as salting-out agents, respectively. It can be seen from Additional file 1: Figure S3a, the recovery of ethanol in K_2_HPO_4_ groups was better than that of K_4_P_2_O_7_ groups. Cations and anions from K_4_P_2_O_7_ could easily associate with ethanol [30], which resulted in a low recovery of ethanol in the upper phase. In Additional file 1: Figure S3b, the acetone recoveries in both two groups remained similar. When the salting-out factor was above 1.25, more than 95.00% of acetone was recovered. For the case of butanol, because of the relatively low polarity index, butanol was much more sensitive to salt. The butanol recoveries in K_4_P_2_O_7_ and K_2_HPO_4_ groups reached 99.31 and 96.38%, respectively, at the salting-out factor of 0.5 (Additional file 1: Figure S3c). Owing to the predominant butanol content in ABE mixture, butanol played a key role in the process of ABE recovery (Additional file 1: Figure S3d).

## Discussion

**Fig. 6:**
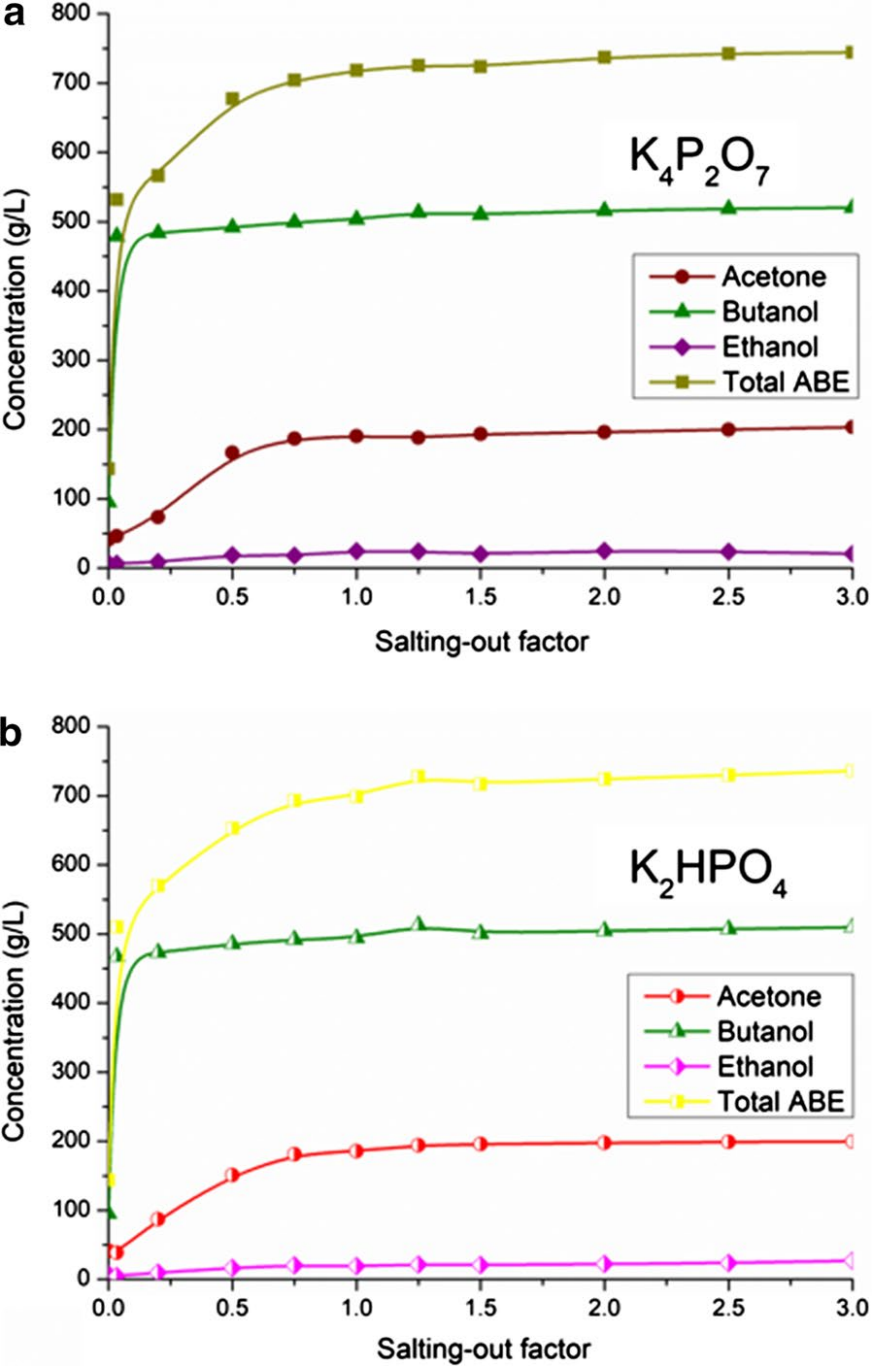
Effect of salting-out factors on ABE concentration in the organic phase. **a** K_4_P_2_O_7_; **b** K_2_HPO_4_

ABE concentrations in the organic phase are determined and the results are shown in Fig. 6. The concentrations of ethanol, acetone, and butanol reached 23.82, 203.46, and 520.30 g/L (total ABE of 747.58 g/L) when the salting-out factor was 3.0, respectively (Fig. 6a). In addition, 23.73, 199.64, and 509.70 g/L (total ABE of 733.08 g/L) were detected in the K_2_HPO_4_ groups (Fig. 6b). Therefore, the two-stage gas stripping–salting-out integration process for in situ ABE recovery was competitive to the previous reports based on two-stage separation processes.

Table 1 summarizes the current advance of in situ recovering high-titer ABE products. Salting-out was an effective method for ABE separation from fermentation broth [26]. However, because the larger volume of the fermentation broth than that of the condensate, it required more salt solution when separating the fermentation broth directly. At the same time, though the salting-out process was simple operation, the contamination of the by-products and cells in broth [26] and the energy-intensive salt recycled also made the process impracticable.

**Table 1:** Current advance in high-titer ABE production based on two-stage ISPR processes

Processes	Substrate	Strains	First-stage separation			Second-stage separation					References
			Butanol in bioreactor (g/L)	Butanol (g/L)	ABE (g/L)	Butanol (g/L)	ABE (g/L)	Ethanol recovery (%)	Acetone recovery (%)	Butanol recovery (%)	
One-stage separation											
Gas stripping	Glucose	*C. acetobutylicum* ABE 1401	14.3	115	180	–	–	–	–	–	[17]
Salting-out	Glucose	–	–	NG	NG	–	–	~ 40^a^	~ 72^a^	~ 100^a^	[26]
Two-stage separation											
Gas stripping–pervaporation	Glucose	*C. acetobutylicum* ABE 1401	10–12	108.3	177.6	482.55	706.68	82.8	99.5	98.8	[12]
	Glucose	*C. acetobutylicum*JB200	16.2	155.6	199.9	441.7	593.2	87.5	81.4	92.2	[9]
Two-stage gas stripping	Glucose	*C. acetobutylicum*JB200	19.2	175.6	227.0	420.3	532.3	99.1	78.9	85.4	[8]
Two-stage pervaporation	Glucose	*C. acetobutylicum* ABE 1401	10–12	199.1	346.5	451.98	782.5	~ 67	~ 100	99.3	[33]
Extraction-gas stripping	Glucose	*C. acetobutylicum* ATCC 824	NG	29–32	37–42	200–250	360–460	NG	NG	88	[32]
Fraction-salting-out	–	–	–	~ 260	~ 400	NG	NG	98.2	100	100	[25]
Gas stripping–salting-out	SSB	*C. acetobutylicum* ABE 1201	8.6–9.4	95.1	143.6	520.3	747.59	89.31	99.12	~ 100	This work

*NG* not given

^a^ The recovery of organic solvent from the first-stage salting-out

To further determine the salt usage and the energy requirement of the salt recycling process, simple technical–economic analysis was further carried out. In the current work, gas stripping condensate (143.6 g/L of ABE) was used for salting-out process, rather than the fermentation broth. That means, to separate certain mass of ABE by salting-out, the volume of the fermentation broth required was 11.8 times higher than the gas stripping condensate (our previous work showed that the ABE concentration in the cellulosic ABE fermentation broth was lower than 12.2 g/L [10]). Hence, the salt requirement for ABE separation from gas stripping condensate was only ~ 10% of the salt usage in the cause of ABE separation from fermentation broth. Although this comparison was very cursory, the difference of ABE concentration, the water contained and many other issues were not considered in this estimation, it was still certain that large amount of salt might be saved because of the large volume reduction of the salting-out agent for given amount of ABE product.

Previous works also demonstrated that the high cost of salt was another key factor that affected the possibility of the salting-out process [34]. In this work, K_4_P_2_O_7_ and K_2_HPO_4_, the two kinds of salt were all commonly used in industry, showing good solubility and salting-out effects. According to our market survey in China, calculation and estimation (~ 1290 $/t for K_4_P_2_O_7_ and ~ 790 $/t for K_2_HPO_4_), under the optimized salting-out condition (at the salting-out factor of 3.0, 323.15 K, the solubility of K_4_P_2_O_7_ and K_2_HPO_4_ was 66.7 wt. and 66.3 wt. %, see Additional file 1: Figure S1), the initial cost of K_4_P_2_O_7_ and K_2_HPO_4_ for 1 kg of ABE production from gas stripping condensate was estimated at ~ 18 and ~ 11 $, respectively. However, these costs were not the actual cost for ABE salting-out. As it showed in the previous studies, the salt in aqueous phase could be recovered by vacuum distillation [25, 27]. Water absorbed in could be recovered by simple evaporation. In addition, the recycled saturated salt solution showed the potential reutilization in the following batches of salting-out. Therefore, salt in the salting-out process is the one-off investment.

We further estimated the evaporation energy cost for water recovery from the diluted salt solution after salting-out process based on process simulation. The evaporation energy required for the conventional salting-out process that feeding the fermentation broth directly was also evaluated as the control group. Results shown in Additional file 1: Figure S4 indicated that compared with the K_2_HPO_4_ group (15.14 MJ/kg of ABE), the energy requirement for salt recycling in the K_4_P_2_O_7_ group was lower. 14.77 MJ/kg of energy was demanded for water evaporation. In contrast, because of the low concentration of ABE in broth and large amount of saturated salt solution required, the evaporation energy cost in the control group was above 197.3 MJ/kg, which was 13.36-time higher than that of the current gas stripping–salting-out process.

Our previous research pointed out that second-stage pervaporation in the two-stage gas stripping–pervaporation separation process was under low efficiency compared with the pervaporation unit that integrated with fermentation system alone [12]. Even so, the energy requirement (evaporation energy, not included the electric energy consumption for recycling the ABE solution and the controlling system) for the second-stage ABE separation by pervaporation was only ~ 13 MJ/kg of ABE (in that process, ABE concentration increased from 119.4 to 706.68 g/L), which was slightly lower than the energy cost of salt recovery process in the current work. Therefore, the current salting-out unit in the second-stage ABE separation was energy-intensive compared with the previous work [12]. However, it was worthy to note here that the estimation of the water evaporation energy cost from the salting-out solution was based on simple vacuum distillation, and other energy-efficient methods such as multiple-effect evaporation process were not simulated and evaluated in the estimation. Thus, we boldly speculated that the salting-out system for the second-stage ABE separation was as effective as the pervaporation process. More importantly, the large amount of low-grade heat source from the downstream distillation process (e.g., streams 4, 8, and 11 showed in Additional file 1: Table S1) could be not only used for warming the bioreactor, compensating the energy requirement in the first-stage gas stripping process, but also showed great potential reutilization in the salt recycling process for water evaporation. Hence, the net heat consumption could be ignored in the salting-out unit after heat exchange. Our next work will focus on the investigation of the heat-exchange and energy-saving of the whole biorefinery system.

The gas stripping–salting-out process also showed superiorities of high ABE titer and high recovery. In addition, because of the pre-concentration of ABE solvents by gas stripping in the first-stage separation, salt regeneration might be easier, because there was no contamination of the by-products and little water content [13]. There was also no need additional condensation in the second-stage separation compared with the previous works using two-stage gas stripping–pervaporation and two-stage pervaporation systems [12, 33]. Thus, the energy consumption for solvents condensation was also saved.

Actually, the salting-out system in the integration process could save more energy as expected in the subsequent downstream distillation. To confirm it, process simulation was carried out by the UNISIM software and the NRTL model. According to our previous work [12], acetone, ethanol, water, and butanol were separated successively based on their different boiling points. Because of the relatively high ABE content in the feeding streams, the organic phase of salting-out systems in two scenarios was all exceed the azeotropic point. Therefore, the beer column of the conventional distillation process was no longer needed [12]. After developing the series of distillation, pinch analysis was carried out to further decrease the overall heat requirement. Detailed parameters of the distillation process were attached in the additional files (Additional file 1: Table S1 shows the mass composition and temperatures of the streams, Additional file 1: Table S2 shows the energy requirement of each column, and Additional file 1: Figure S5 shows the ABE distillation process based on the salting-out process using K_4_P_2_O_7_ and K_2_HPO_4_, respectively).

Results indicated that both the two scenarios based on different salt contents showed promising in decreasing the energy requirement of downstream distillation process. 3.68 MJ/kg ABE of heat was required to separate high-purity products (100 wt. % of butanol, 99.7 wt. % of acetone, and 95 wt. % of ethanol) from the organic phase in the K_2_HPO_4_ group, while the energy requirement in the K_4_P_2_O_7_ group was 3.72 MJ/kg. The higher energy cost of the K_4_P_2_O_7_ group was caused by the higher energy requirement in the acetone and butanol columns (Additional file 1: Table S2 and Figure S5). However, in consideration of the higher ABE recovery of the K_4_P_2_O_7_ group (Fig. 5) and the lower energy requirement in the recovery process of K_4_P_2_O_7_ solution (Additional file 1: Figure S4), the slight difference of the distillation energy cost between the two scenarios was negligible. In addition, it was easy to draw the conclusion that the K_4_P_2_O_7_ agent was the better choice for solvents separation in the current novel two-stage gas stripping–salting-out process for ABE production.

In the literatures, 98.8 and 99.3% (w/v) of butanol were recovered after the second-stage pervaporation [12, 33]. In these processes, the second-stage pervaporation process was complex and had little effect on the reduction of energy cost. In comparison with this, the current two-stage gas stripping–salting-out process provided higher butanol and by-products (acetone and ethanol) recoveries. It was also worthy to note that the residual acetone and ethanol in the aqueous phase were negligible (86.95% of acetone and 70.09% of ethanol were recovered after salting-out by K_4_P_2_O_7_). However, because of the relatively low ethanol content in retentate and polarity difference between ethanol molecular and the active layer of pervaporation membrane, only ~ 67% (w/v) of ethanol was recovered in the two-stage pervaporation series [33]. Therefore, the salting-out process showed advantage in higher solvents recovery, which improved the carbon atom economy in the novel integration process.

## Conclusions

Cellulosic ABE production was effectively recovered by gas stripping–salting-out integration process. Compared with K_2_HPO_4_, K_4_P_2_O_7_ was more effective in the second-stage salting-out system. ABE concentration increased progressively from 14 to 15 g/L in the fermentation broth to 143.6 g/L in the gas stripping condensate after the first-stage separation, and it reached 747.58 g/L after the second-stage salting-out. High ABE recovery of 99.32% was achieved under the optimum condition (the salting-out factor was 3.0 and the temperature of saturated K_4_P_2_O_7_ solution was 323.15 K). Only 3.72 MJ/kg of ABE was required in the downstream distillation process. The novel integration process was attractive for effective ABE separation from fermentation broth.

## Additional file

**Additional file 1: Figure S1.** The solubility of K_4_P_2_O_7_ or K_2_HPO_4_ in water at given temperature, **Figure S2.** Comparison of solvents concentration remained in the aqueous phase; **Figure S3.** Comparison of solvents recovery in the organic phase using different types of salting-out agents. **Figure S4.** Evaporation energy requirement for water removal from the agents after salting out. **Table S1.** Temperatures and the mass composition of the streams in the distillation processes. **Table S2.** Comparison of the energy cost of the distillation process before and after heat exchange. **Figure S5.** Downstream distillation process using the organic phase after two-stage gas stripping–salting-out as feed.

## Abbreviations

ABE: acetone–butanol–ethanol
ISPR: in situ product recovery
SSB: sweet sorghum bagasse
FID: flame ionization detector
RID: differential refraction detector
GC: gas chromatograph
