# Supplementary material for: Integrated in situ gas stripping–salting-out process for high-titer acetone–butanol–ethanol production from sweet sorghum bagasse
Source: Biotechnol Biofuels. 2018 May 10;11:134. doi: 10.1186/s13068-018-1137-5 (PMC5944105; doi:10.1186/s13068-018-1137-5)
Supplement: Supplementary file 1 — Additional file 1: Figure S1. The solubility of K4P2O7 or K2HPO4 in water at given temperature, Figure S2. Comparison of solvents concentration remained in the aqueous phase; Figure S3. Comparison of solvents recovery in the organic phase using different types of salting-out agents. Figure S4. Evaporation energy requirement for water removal from the agents after salting out. Table S1. Temperatures and the mass composition of the streams in the distillation processes. Table S2. Comparison of the energy cost of the distillation process before and after heat exchange. Figure S5. Downstream distillation process using the organic phase after two-stage gas stripping–salting-out as feed. [file 13068_2018_1137_MOESM1_ESM.docx]

**Additional files**

Integrated *in situ* gas stripping-salting-out process for high-titer acetone-butanol-ethanol production from sweet sorghum bagasse

Hao Wen^a,b^, Huidong Chen^c,d^, Di Cai^a,b^*, Peiwen Gong ^b^, Tao Zhang ^b^, Zhichao Wu ^b^, Heting Gao ^b^, Zhuangzhuang Li ^c^, Peiyong Qin^a,b^*, Tianwei Tan^a,b^

^a^ National Energy R&D Center for Biorefinery, Beijing University of Chemical Technology, Beijing 100029, PR China

^b^ College of Life Science and Technology, Beijing University of Chemical Technology, Beijing 100029, PR China

^c^ College of Chemical Engineering, Beijing University of Chemical Technology, Beijing 100029, PR China

^d^ Center for Process Simulation & Optimization, Beijing University of Chemical Technology, Beijing 100029, PR China

* Corresponding authors

Address: No.15 Beisanhuan East Road, Chaoyang district, Beijing, 100029.

Email: caidibuct@163.com; qinpeiyong@tsinghua.org.cn

**Fig.S1** The solubility of K_4_P_2_O_7_ or K_2_HPO_4_ in water at given temperatures (298.15 K, 310.65 K and 323.15 K). They were tested in laboratory based on the method of turbidity point. 310.65 K was included because it was similar to the optimized temperature of ABE fermentation.





**Fig.S2** Comparison of solvents concentration remained in the aqueous phase using different types of salting-out agents. (a) Ethanol; (b) acetone; (c) butanol; (d) total ABE.


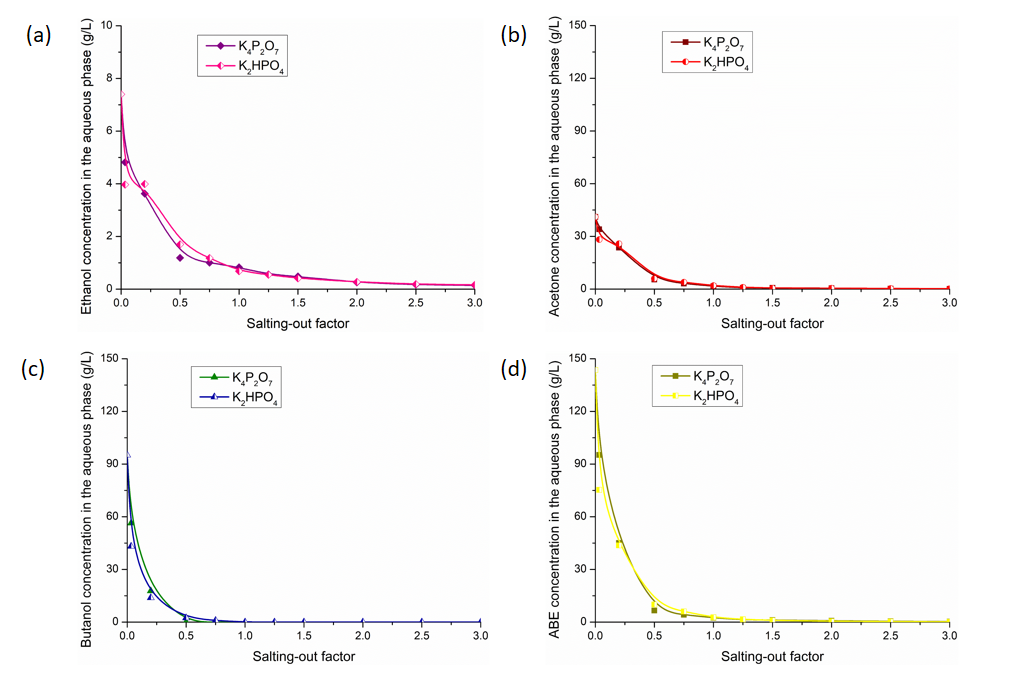


**Fig.S3** Comparison of solvents recovery in the organic phase using different types of salting-out agents. (a) Ethanol; (b) acetone; (c) butanol; (d) total ABE.


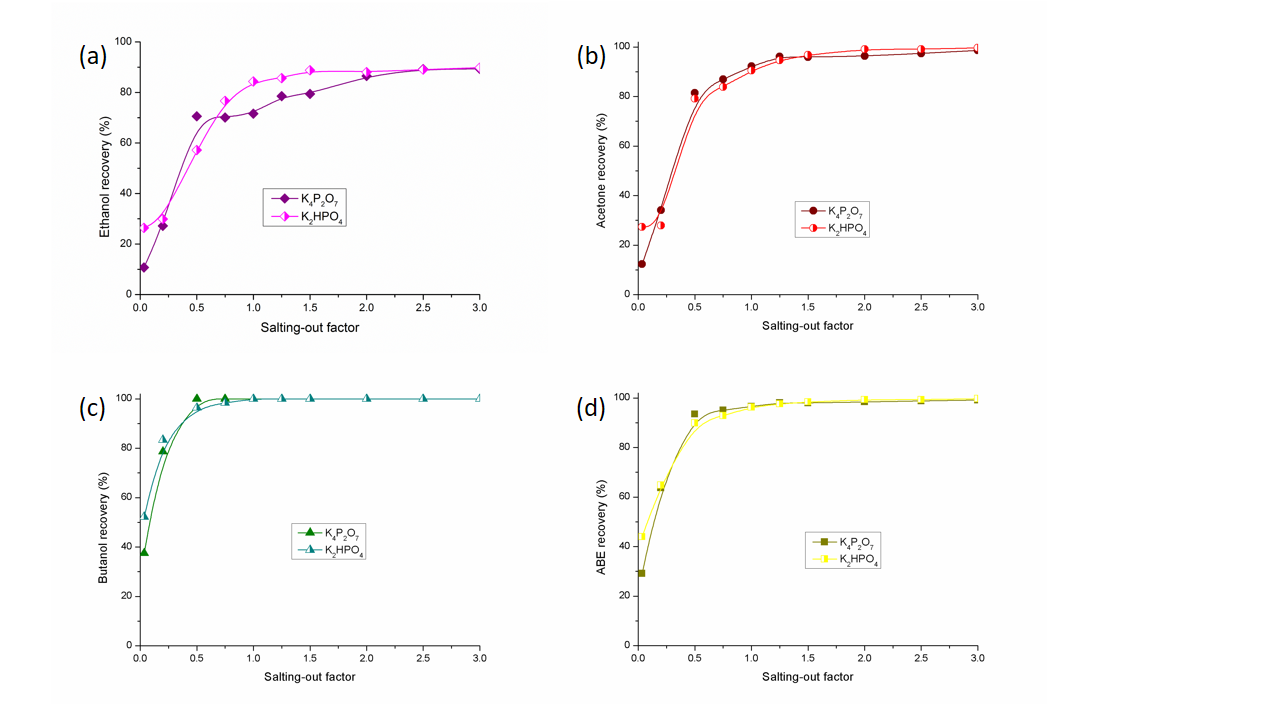


**Fig.S4** Evaporation energy requirement for water removal from the agents after salting out. Salt recycling after ABE salting-out from fermentation broth was treated as the control group. It was clear that the theoretical evaporation energy requirement in the conventional salting-out process based on the fermentation broth was far higher than that of the two-stage gas stripping-salting-out process. And the group using K_4_P_2_O_7_ showed lower energy requirement compared with the K_2_HPO_4_ group in the salt recycling system.





**Fig.S5** Downstream distillation process using the organic phase after two-stage gas stripping-salting-out as feed. 1000 kg/h of the flow was simulated and summarized in two scenarios. The column pressure maintained similar (at atmospheric pressure). And heat exchange was carried out based on pinch analysis. (a) The K_4_P_2_O_7_ scenario; (b) the K_2_HPO_4_ scenario.


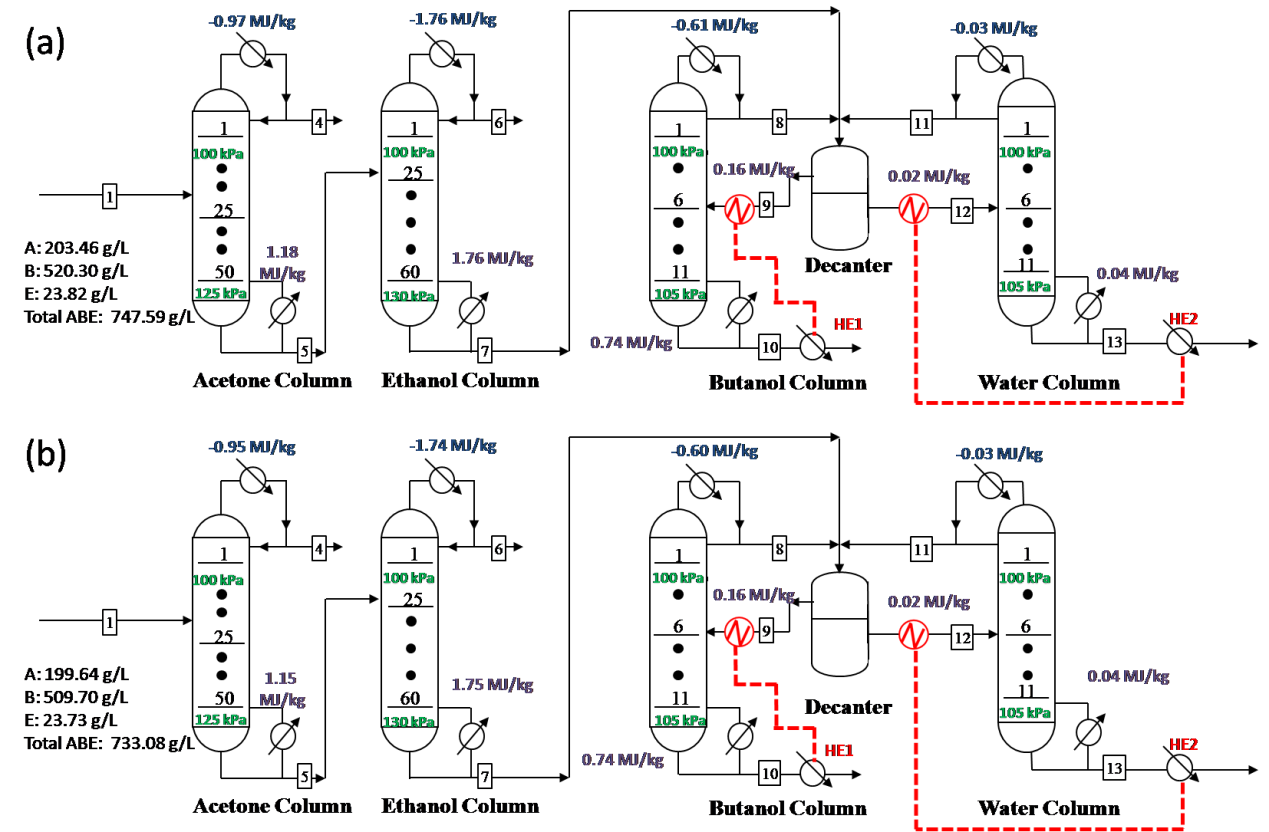


**Table S1**Temperatures and the mass composition of the streams in the distillation processes feeding the organic phase after two-stage gas stripping-salting-out.

| No. | Stream | Acetone (wt.%) | | Ethanol (wt.%) | | Butanol (wt.%) | | Water (wt.%) | | Temperature (^o^C) | |
| --- | --- | --- | --- | --- | --- | --- | --- | --- | --- | --- | --- |
|  |  | K_4_P_2_O_7_ | K_2_HPO_4_ | K_4_P_2_O_7_ | K_2_HPO_4_ | K_4_P_2_O_7_ | K_2_HPO_4_ | K_4_P_2_O_7_ | K_2_HPO_4_ | K_4_P_2_O_7_ | K_2_HPO_4_ |
| 1 | Organic phase after salting-out | 24.95 | 24.37 | 63.8 | 62.22 | 2.92 | 2.9 | 8.33 | 10.5 | 50 | 50 |
| 4 | Distillate of acetone column | 99.7 | 99.7 | 0.1 | 0.1 | 0 | 0 | 0.2 | 0.2 | 55.7 | 55.7 |
| 5 | Bottom output of acetone column | 0 | 0 | 3.86 | 3.8 | 85.09 | 82.36 | 11.05 | 13.84 | 100.1 | 100.1 |
| 6 | Distillate of ethanol column | 0 | 0 | 95 | 95 | 0 | 0 | 5 | 5 | 77.77 | 77.77 |
| 7 | Bottom output of ethanol column | 0 | 0 | 0 | 0 | 88.7 | 85.8 | 11.3 | 14.2 | 103.3 | 102.1 |
| 8 | Distillate of butanol column | 0 | 0 | 0 | 0 | 27.98 | 28.32 | 72.08 | 71.68 | 92.65 | 92.66 |
| 9 | Organic phase of decanter | 0 | 0 | 0 | 0 | 85.17 | 85.17 | 14.83 | 14.83 | 20 | 20 |
| 10 | Bottom output of butanol column | 0 | 0 | 0 | 0 | 99.99 | 99.99 | 0.01 | 0.01 | 118.8 | 118.8 |
| 11 | Distillate of water column | 0 | 0 | 0 | 0 | 19.09 | 23.49 | 80.91 | 76.51 | 91.42 | 91.75 |
| 12 | Aqueous phase of decanter | 0 | 0 | 0 | 0 | 2.5 | 2.5 | 97.5 | 97.5 | 20 | 20 |
| 13 | Bottom output of water column | 0 | 0 | 0 | 0 | 0 | 0 | 100 | 100 | 101 | 101 |

**Table S2** Comparison of the energy cost of the distillation process after the gas stripping-salting-out process before and after heat exchange.

| Column | Distillation based on K_4_P_2_O_7_ | | Distillation based on K_2_HPO_4_ | |
| --- | --- | --- | --- | --- |
|  | Before heat exchange | After heat exchange | Before heat exchange | After heat exchange |
| Acetone column | 1.18MJ/kg | 1.18 MJ/kg | 1.15 MJ/kg | 1.15 MJ/kg |
| Ethanol column | 1.76 MJ/kg | 1.76 MJ/kg | 1.75 MJ/kg | 1.75 MJ/kg |
| Butanol column | 0.90 MJ/kg | 0.74 MJ/kg (0.16 MJ/kg was from HE1) | 0.90 MJ/kg | 0.74 MJ/kg (0.16 MJ/kg was from HE1) |
| Water column | 0.06 MJ/kg | 0.04 MJ/kg (0.02 MJ/kg was from HE2) | 0.06 MJ/kg | 0.04 MJ/kg  (0.02 MJ/kg was from HE2) |
| Total energy consumption | 3.90MJ/kg | 3.72 MJ/kg | 3.86 MJ/kg | 3.68MJ/kg |
